# Supplementary material for: The Crystal Structure of the Defense Conferring Rice Protein OsJAC1 Reveals a Carbohydrate Binding Site on the Dirigent-like Domain
Source: Biomolecules. 2022 Aug 17;12(8):1126. doi: 10.3390/biom12081126 (PMC9405769; doi:10.3390/biom12081126)
Supplement: Supplementary file 1 [file biomolecules-12-01126-s001.zip › biomolecules-1825139-supplementary.pdf]

The Crystal Structure of the Defense Conferring Rice Protein *Os*JAC1 Reveals a Carbohydrate Binding Site on the Dirigent-like Domain

Nikolai Huwa [1], Oliver H. Weiergräber [2], Alexander V. Fejzagić [1], Christian Kirsch [3], Ulrich Schaffrath [3] and Thomas Classen [4,*]

[1] Institute for Bioorganic Chemistry, Heinrich Heine University Düsseldorf, 52425 Jülich, Germany
[2] Institute of Biological Information Processing 7: Structural Biochemistry and Jülich Centre for Structural Biology, Forschungszentrum Jülich, 52425 Jülich, Germany
[3] Institute for Biology III, Department of Plant Physiology, RWTH Aachen University, 52056 Aachen, Germany
[4] Institute for Bio- and Geosciences 1: Bioorganic Chemistry, Forschungszentrum Jülich, 52425 Jülich, Germany
* Correspondence: t.classen@fz-juelich.de

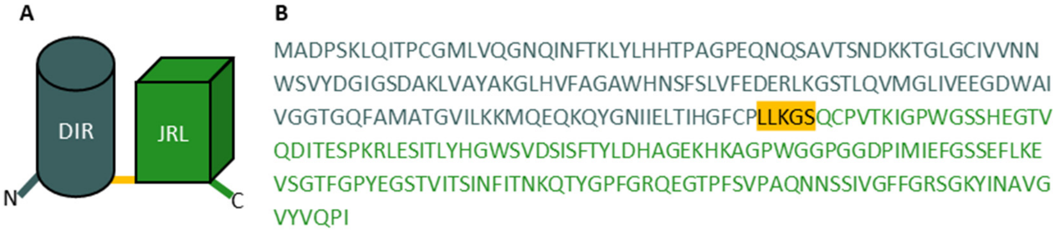

Figure S1. Domain arrangement of *Os*JAC1. A) Schematic domain arrangement of *Os*JAC1 with the DIR domain (metallic) at the N-terminus and the JRL domain (green) at the C-terminus. The linkage of both domains is highlighted in yellow. B) Amino acid sequence of *Os*JAC1, with domains coloured as in A.

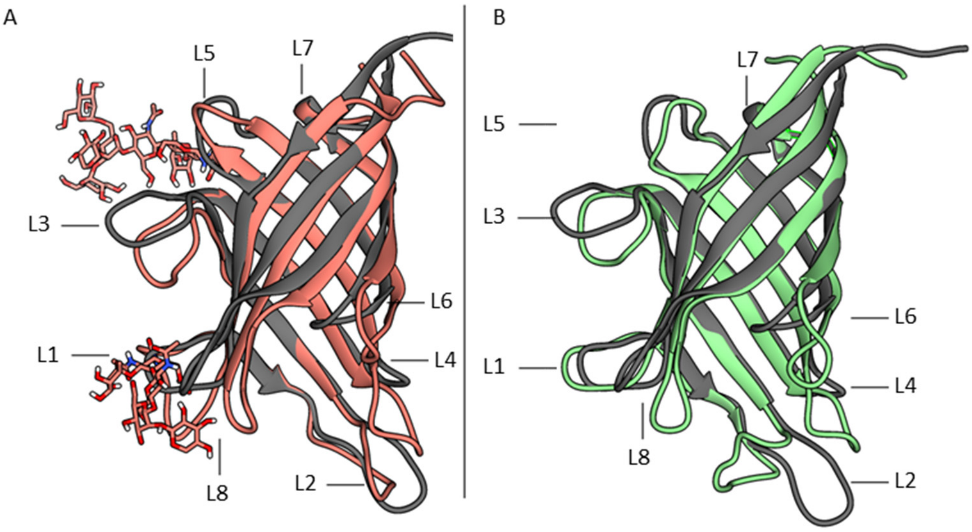

Figure S2. Structural comparison of *Os*JAC1-DIR with other dirigent proteins. Structure alignment of the *Os*JAC1-DIR domain (cf1, chain A, grey) in ribbon representation with A) *At*DIR6 including glycosylation at Asn59 und Asn123 (red; PDB: 5LAL) and B) *Ps*PTS1 (green; PDB: 5OOD). Loops of *Os*JAC1-DIR are labelled L1 through L8.

Table S1. *Os*JAC1 residues selected as "active" for protein-protein docking in HADDOCK [44].

Molecular entity	Selected residues for HADDOCK docking
DIR (docking to JRL)	42, 43, 44, 45, 46, 47, 48, 49, 50, 51, 52, 53, 54, 55, 56, 58, 60, 78, 79, 80, 82, 83, 84, 85, 86, 87, 88, 90, 92, 94, 98, 101, 102, 106, 108, 109, 110, 111, 115, 116, 118, 120, 121, 122, 123, 124, 125, 127, 128, 130, 131, 132, 134, 152, 153, 156
JRL (docking to DIR)	160, 175, 176, 177, 178, 179, 180, 181, 182, 206, 208, 209, 210, 211, 212, 213, 214, 216, 217, 219
JRL (dimerization of two-domain model)	160, 161, 162, 163, 164, 165, 166, 167, 273, 274, 276, 277, 278, 280
DIR (dimerization of two-domain model)	42, 43, 44, 45, 46, 47, 48, 49, 50, 51, 52, 53, 54, 55, 56, 58, 60, 78, 79, 80, 82, 83, 84, 85, 86, 87, 88, 90, 92, 94, 98, 101, 102, 106, 108, 109, 110, 111, 115, 116, 118, 120, 121, 122, 123, 124, 125, 127, 128, 130, 131, 132, 134, 152, 153, 156

Table S2. Scoring results of HADDOCK.

Docking results	DIR-JRL (Figure 6)	Type-I dimer (Figure 7A)	Type-II dimer (Figure 7B)
HADDOCK score	-45.1 +/- 7.5	-98.3 +/- 1.6	-154.5 +/- 1.5
Cluster size	7	183	29
RMSD from the overall lowest-energy structure [Å]	17.4 +/- 0.1	0.7 +/- 0.4	0.6 +/- 0.4
Van der Waals energy [kJ/mol]	-58.7 +/- 6.2	-68.8 +/- 1.7	-69.4 +/- 4.8
Electrostatic energy [kJ/mol]	-120.5 +/- 23.2	-60.4 +/- 8.8	-540.3 +/- 23.5
Desolvation energy [kJ/mol]	-4.4 +/- 2.0	-18.4 +/- 2.4	5.8 +/- 2.3
Restraints violation energy [kJ/mol]	421.2 +/- 49.9	9.0 +/- 3.0	171.7 +/- 26.8
Buried Surface Area [Å^2]	1892.5 +/- 87.6	1744.2 +/- 29.0	3220.1 +/- 16.4
Z-Score	0.0	-1.4	-1.1

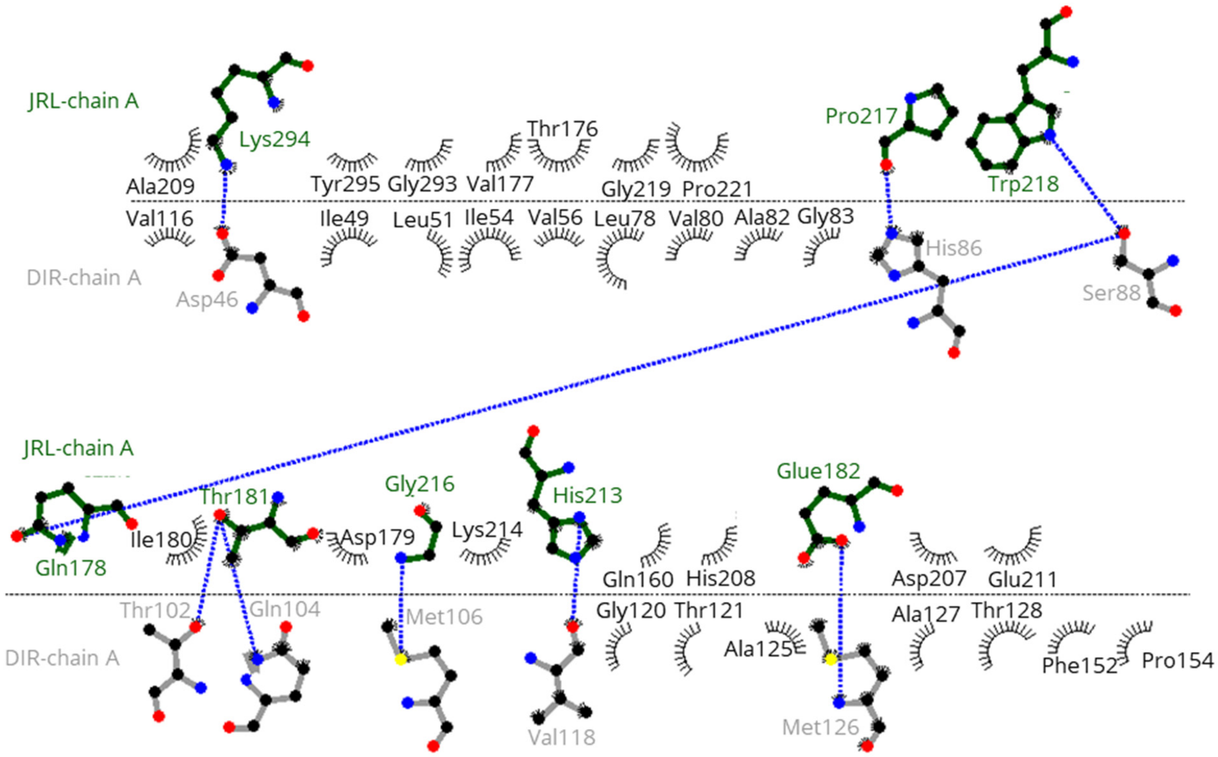

Figure S3. Dimer interaction of the DIR-JRL-complex. DIMPLOT representation [41] of the DIR-JRL complex model, including residues involved in hydrophobic interaction (black) and hydrogen bond interaction (blue lines). Nitrogen, oxygen, and sulphur atoms are coloured blue, red, and yellow, respectively. The horizontal dashed line represents the interface between the two domains.

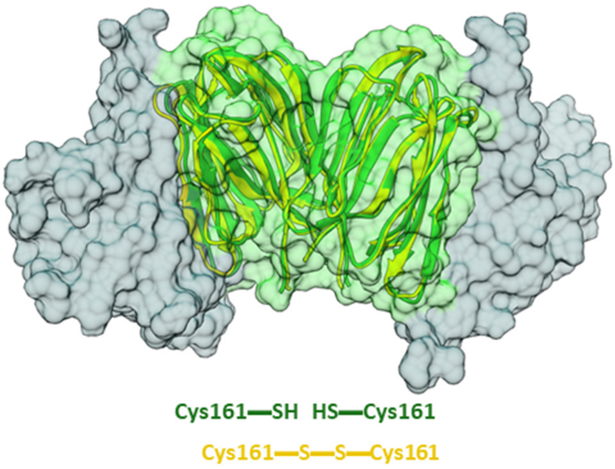

Figure S4. Structural alignment of the proposed JRL-mediated *Os*JAC1 dimer; (showing the JRL and DIR domains as green ribbon and metallic surface, respectively) and the JRL homo-dimer derived from the crystal structure of JRL-cf1 (yellow ribbon) The corresponding oxidation states of JRL residue Cys161 are indicated.

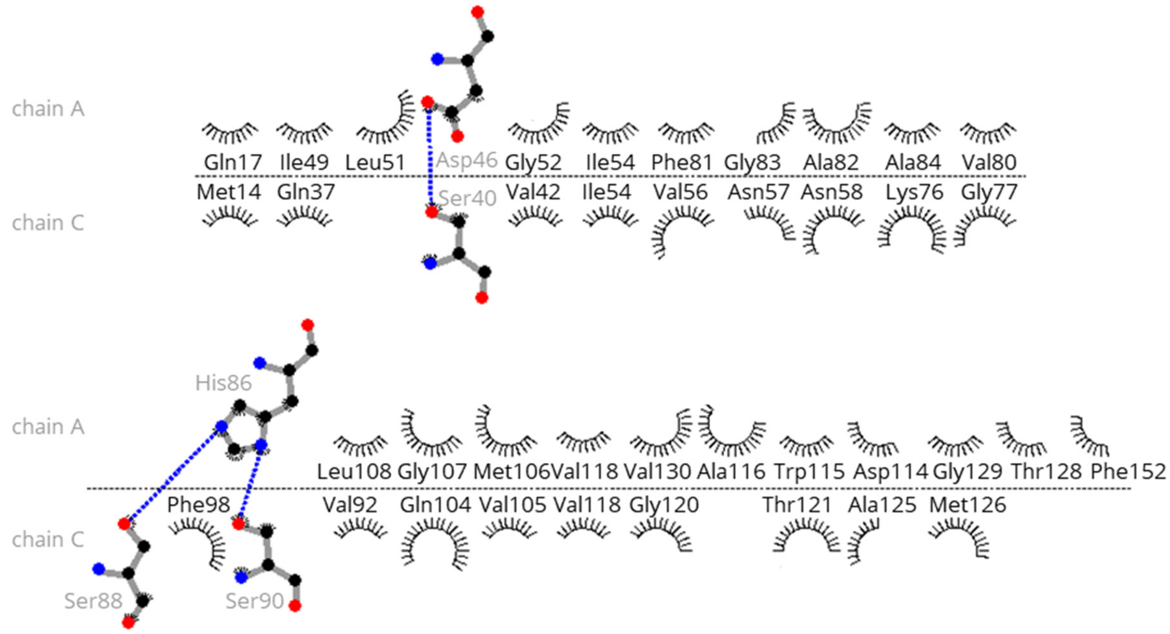

Figure S5. Trimer interaction of the DIR-DIR-complex. DIMPLOT representation [41] of the binary -DIR-DIR interface within the *Os*JAC1-DIR homo-trimer (cf1, chains A and C), including residues involved in hydrophobic interaction (black) and hydrogen bond interaction (blue lines). Nitrogen, oxygen, and sulphur atoms are coloured blue, red, and yellow, respectively. Interactions between all pairs of chains are similar if residues preceding Leu26 or following Cys153 are omitted.

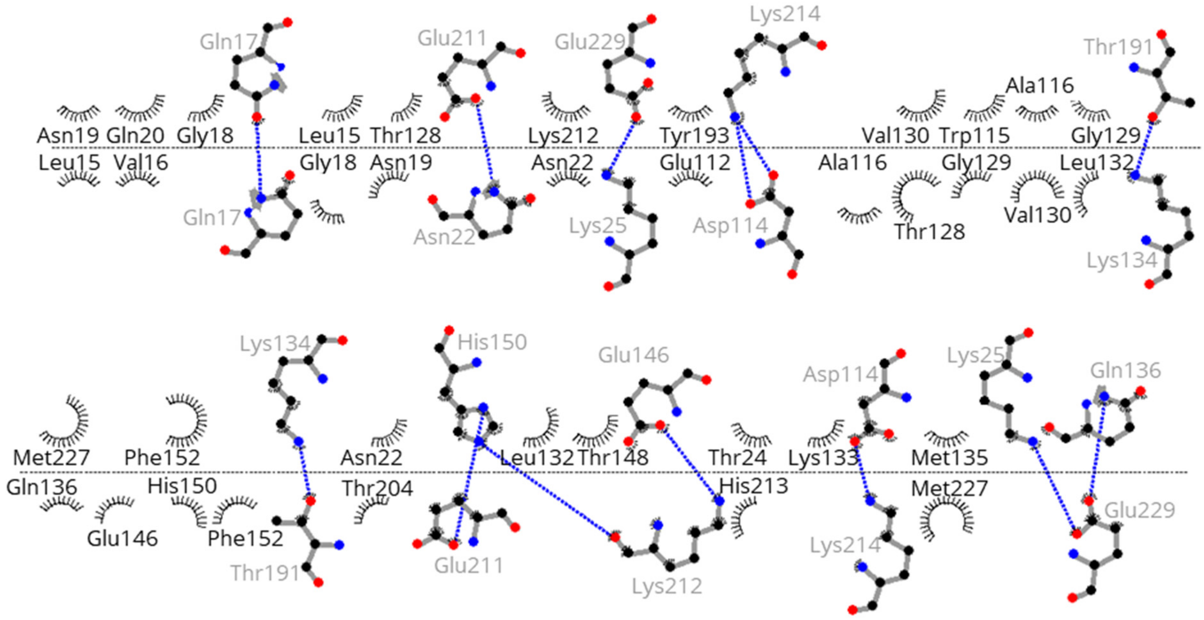

Figure S6. Dimer interaction of the DIR-DIR-complex. DIMPLOT representation [41] of a DIR-guided *Os*JAC1 dimerization mode featuring an interface between the DIR domains (14-155) with additional contributions from the JRL domains (160-306). Residues involved in hydrophobic interaction (black) and hydrogen bond interaction (blue lines) are indicated, with nitrogen, oxygen, and sulphur atoms coloured blue, red, and yellow, respectively.

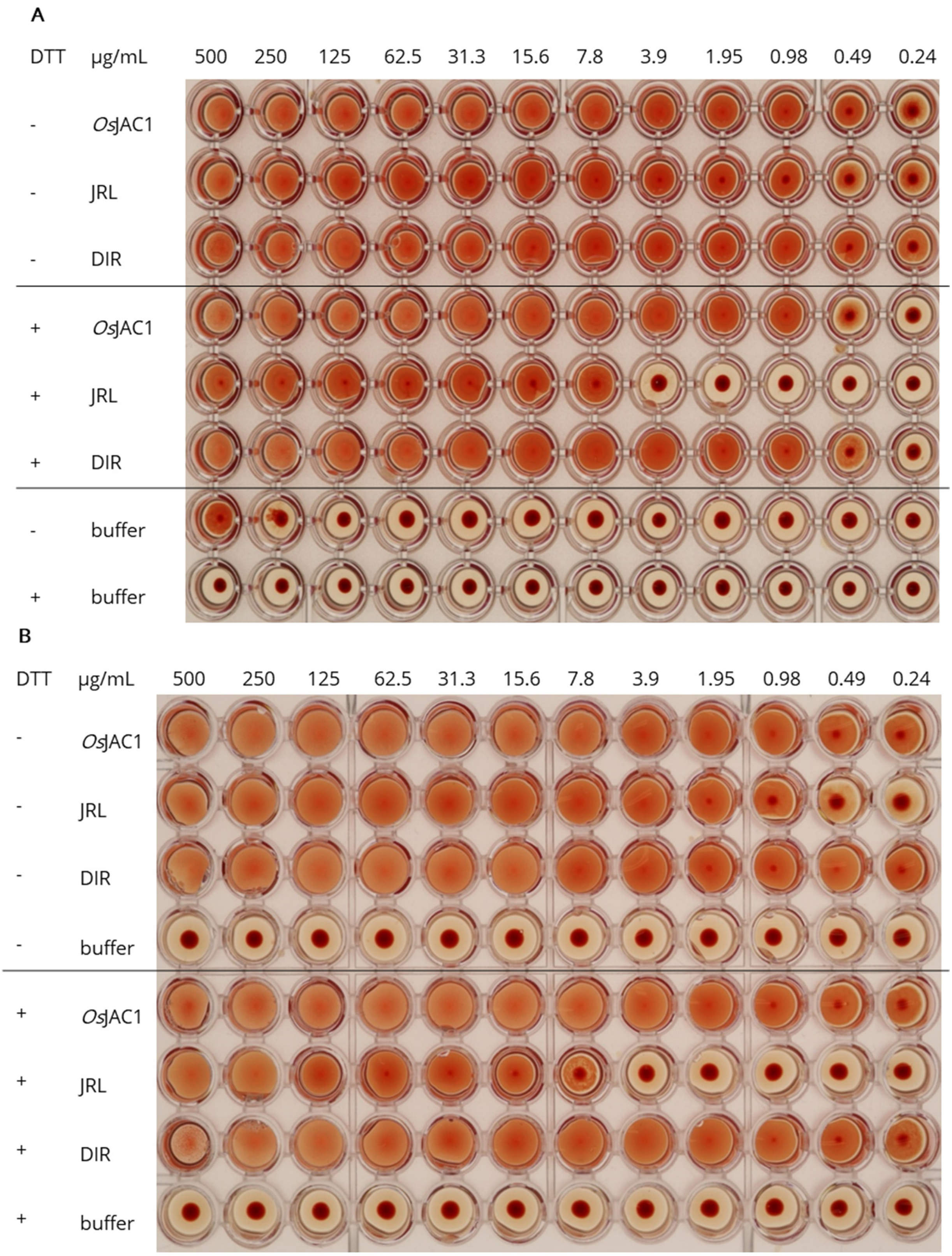

Figure S7. Hemagglutination assay. (A) and (B) Plates of rabbit erythrocytes hemagglutination assay with *Os*JAC1 and its domains (JRL and DIR) under oxidizing (0 mM DTT) or reducing (4 mM DTT) assay conditions Proteins were serially diluted from 500 to 0.24 µg/mL. In the absence of agglutinating agents, erythrocytes sediment to the V-shape bottom of the well, appearing as a dense red spot, whereas a red cloudy mat indicates hemagglutination. The negative controls were the specific buffer conditions without any protein. The *Os*JAC1

JRL domain was used as positive control since it has been shown previously to agglutinate rabbit erythrocytes [11].

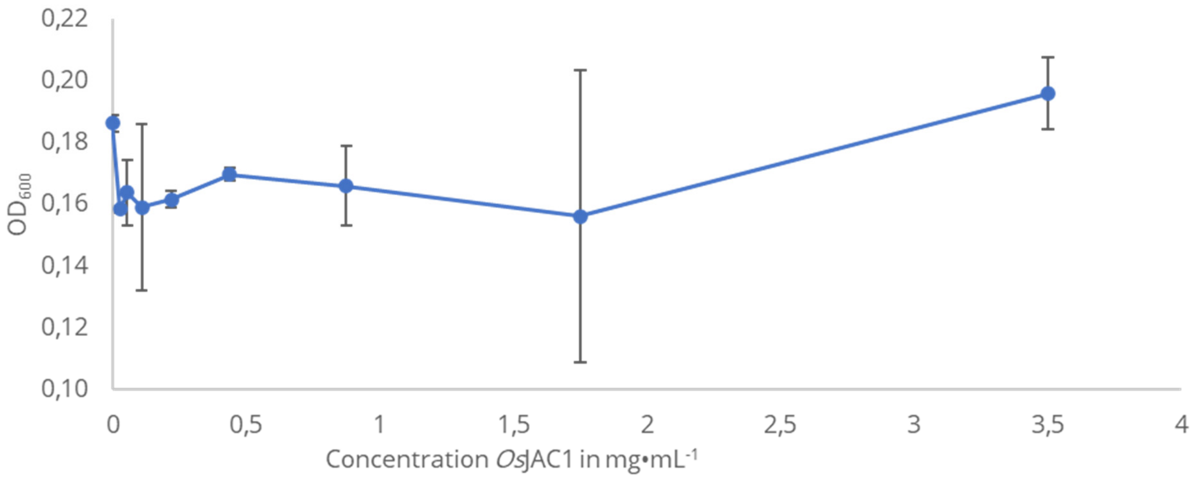

Figure S8. Growth inhibition assay using *Escherichia coli* BL21(DE3) with heterologously produced *Os*JAC1.

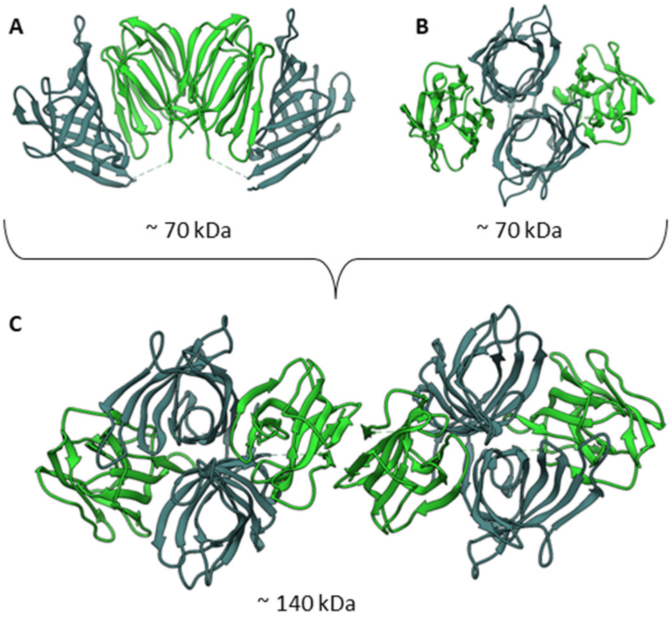

Figure S9. Possible formation of an *Os*JAC1 tetramer under reducing conditions. A) Proposed *Os*JAC1 dimer stabilised by interactions between the JRL domains. B) Proposed *Os*JAC1 dimer stabilised (mostly) by interactions between the DIR domains. C) Combination of both dimerization modes, leading to a tetramer with a molecular mass of approx. 140 kDa.

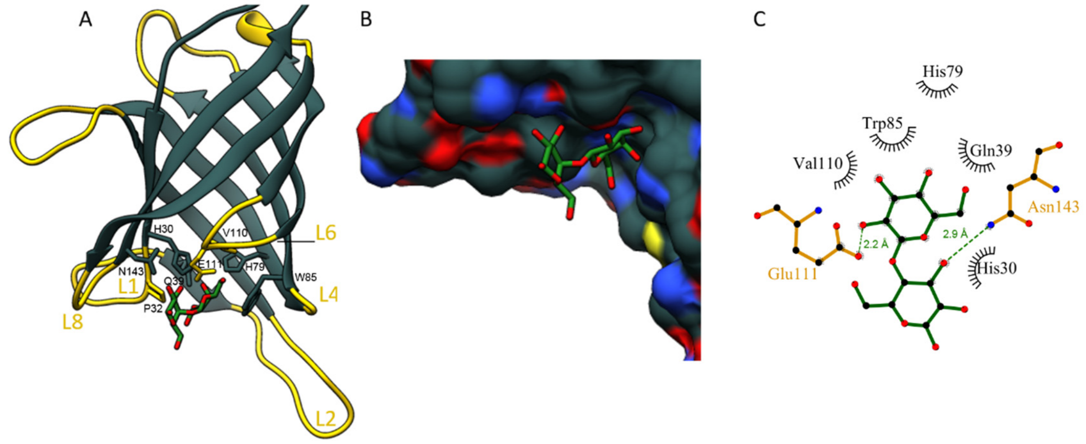

Figure S10. Binding site of galactobiose on the DIR domain of *Os*JAC1. A) Ribbon view of a single DIR domain (chain A). The binding site of galactobiose (stick model with carbon coloured green) is surrounded by the loops L1, L2, L4, L6, and L8 (all colored yellow). B) Molecular surface view of the binding site, oriented similar to panel A and with colored heteroatoms (O red, N blue, S yellow) C) Ligand-protein interaction scheme (generated with LigPlot+ [36] using chain A), illustrating the residues involved in hydrogen bonding (yellow residues) or hydrophobic interaction (black residues).

References

11. Jiang, J.-F.; Han, Y.; Xing, L.-J.; Xu, Y.-Y.; Xu, Z.-H.; Chong, K. Cloning and expression of a novel cDNA encoding a mannose-specific jacalin-related lectin from *Oryza sativa*. *Toxicon* **2006**, *47*, 133-139.

36. Laskowski, R.A.; Swindells, M.B. LigPlot+: Multiple Ligand–Protein Interaction Diagrams for Drug Discovery. *J. Chem. Inf. Model.* **2011**, *51*, 2778–2786, doi:10.1021/ci200227u.

41. Gasper, R.; Effenberger, I.; Kolesinski, P.; Terlecka, B.; Hofmann, E.; Schaller, A. Dirigent protein mode of action revealed by the crystal structure of AtDIR6. *Plant Physiol.* **2016**, *172*, 2165–2175.

44. Kabsch, W.; Sander, C. Dictionary of protein secondary structure: Pattern recognition of hydrogen-bonded and geometrical features. *Biopolymers* **1983**, *22*, 2577–2637, https://doi.org/10.1002/bip.360221211.
